# Preparation, characterization, and in vitro cytogenotoxic evaluation of a novel dimenhydrinate-β-cyclodextrin inclusion complex

**DOI:** 10.17305/bb.2024.10507

**Published:** 2024-12-01

**Authors:** Lamija Hindija, Jasmina Hadžiabdić, Anja Haverić, Ognjenka Rahić, Maida Hadžić Omanović, Lejla Čaluk Klačar, Irma Durmišević, Amina Tucak-Smajić, Merima Šahinović, Edina Vranić

**Affiliations:** 1 Department of Pharmaceutical Technology, Faculty of Pharmacy, University of Sarajevo, Sarajevo, Bosnia and Herzegovina; 2 Laboratory for Cytogenetics and Genotoxicology, Institute for Genetic Engineering and Biotechnology, University of Sarajevo, Sarajevo, Bosnia and Herzegovina

**Keywords:** Dimenhydrinate, β-cyclodextrin, phase solubility, FTIR, DSC, MTT assay, alkaline comet assay, CBMN-cyt

## Abstract

Dimenhydrinate (DMH), used to alleviate motion sickness symptoms, such as nausea, vomiting, dizziness, and vertigo, encounters limitations in oral pharmaceutical formulations due to its poor water solubility and bitter taste. Our research hypothesized that inclusion complexation with β-cyclodextrin (β-CD) might address these drawbacks while ensuring that the newly formed complexes exhibit no cytotoxic or genotoxic effects on peripheral blood mononuclear cells (PBMCs). Inclusion complexes were prepared using the kneading method and the solvent evaporation method. The phase solubility analysis, attenuated total reflectance-Fourier transform infrared spectroscopy (ATR-FTIR), and differential scanning calorimetry (DSC) were conducted to evaluate the complexation efficacy and stability constant of the new binary systems. The results demonstrated that both methods provided complete and efficient complexation. Cytogenotoxic analysis, including the 3-[4,5-dimethylthiazol-2-yl]-2,5-diphenyl tetrazolium bromide (MTT) assay, alkaline comet assay, and cytokinesis-block micronucleus cytome (CBMN-cyt) assay, was conducted to assess the cytogenotoxic potential of DMH-β-CD inclusion complexes, a topic previously unexamined. No cytotoxic or genotoxic effects were observed within the concentration range of 36.36–109.09 ng/mL. Cell viability of treated PBMCs exceeded 85% for all tested concentrations. No significant increases in DNA strand breaks were observed at any dose, and the tail intensity of all complexes remained lower or up to 2.2% higher than the negative control. Parameters indicating genotoxic effects, as well as cytotoxic and cytostatic potential in the CBMN-cyt assay, did not significantly differ from untreated controls. These results suggest that inclusion complexation with β-CD might be a safe and promising solution to overcome the limitations of poor solubility and unpleasant taste of DMH, potentially providing opportunities for new and improved oral pharmaceutical dosage forms.

## Introduction

**Figure 1.**
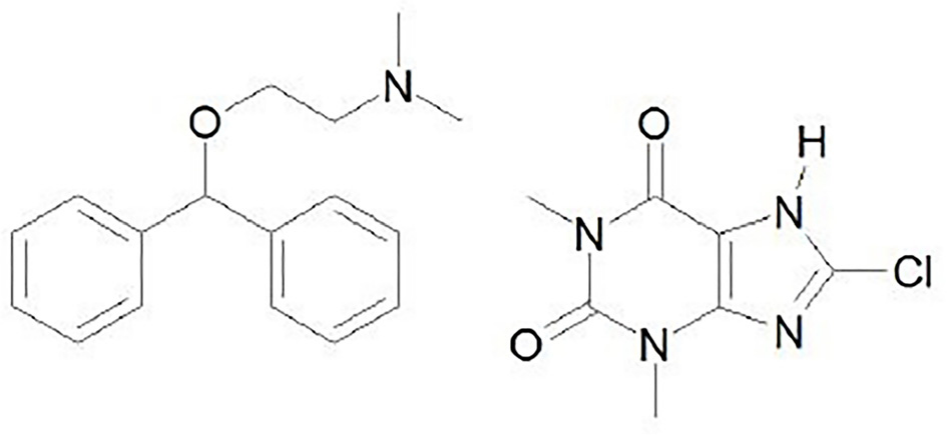
Chemical structure of dimenhydrinate (diphenhydramine on the left and 8-chlorotheophylline on the right) [43].

Almost a third of the world’s population is highly prone to motion-caused nausea, vomiting, dizziness, and vertigo. Motion sickness is induced by a sensory mismatch, a syndrome triggered by a contradiction between the sensory system of balance and vision [1], and it can significantly affect one’s general health, performance, and quality of life [2]. Dimenhydrinate (DMH) is an over-the-counter drug used for the prevention and treatment of these conditions [3]. Comprising two drugs, diphenhydramine (2-diphenylmethoxy-N,N-dimethylethylamine) and 8-chlorotheophylline (Figure 1), DMH engages in dual action. Diphenhydramine acts as the primary antiemetic, alleviating neural excitation through H1 receptor antagonism, while 8-chlorotheophylline counteracts diphenhydramine’s sedative effects by blocking adenosine A2 receptors [4]. DMH is a white, odorless, and bitter crystalline powder, classified as a slightly soluble drug with a logarithm of the partition coefficient (logP) of 3.65 and a molar mass of 470 g/mol. It belongs to class II of the biopharmaceutical classification system (BCS) as a drug with low solubility and high permeability [5, 6]. A peak plasma concentration (C_max_) of 72.6 ng/mL is reached after oral administration of 50 mg [7].

The growing interest in designing new drug-delivery systems stems from the necessity to enhance existing therapies for individual patient needs. To expedite the process and minimize costs associated with new drug development, attention has shifted toward improving pharmaceutical formulations using familiar active pharmaceutical ingredients (APIs) with established safety profiles. Cyclodextrins (CDs) have emerged as effective auxiliary substances for modifying undesirable physicochemical properties of known APIs, facilitating the development of advanced drug-delivery systems [8].

CDs, cyclic oligosaccharides with a hydrophilic outer surface and a lipophilic inner cavity, consist of 6–12 glucose units linked cyclically by α-1,4 glycosidic bonds. They form inclusion complexes by incorporating drug molecules (“guests”) into the internal CD cavity (“hosts”). Established non-covalent bonds improve guest molecules’ physicochemical properties without necessitating chemical changes that are important for their therapeutic profiles. Inclusive complexation provides a solubility increment of poorly soluble drugs (BCS class II or IV), ultimately increasing the dissolution rate, bioavailability, and release profile, and might also mask the guest’s unpleasant taste or smell [8, 9].

CD complexation enhances the oral bioavailability of poorly water-soluble drugs with high lipophilicity (logP > 2.5), administered at low doses (< 100 mg) with DMH being an optimal candidate for complexation [10], due to its low solubility (3 mg/mL) [5], high lipophilicity (logP ═ 3.65) [3], and low dosage (25 or 50 mg in oral formulations) [11]. Its suitability is attributed to meeting specific requirements, including a skeleton with more than five carbon (C) and nitrogen (N) atoms, only two condensed rings, a melting point below 250 ^∘^C, hydrophilic functional groups, and less than five condensed rings [10].

**Figure 2.**
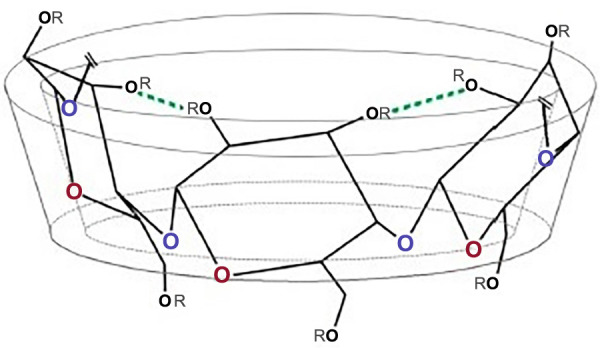
**Structure of β-cyclodextrin molecule.** Green lines represent intramolecular hydrogen bonds that are formed when there are no hydrophilic substituents [83].

β-cyclodextrin (β-CD) is readily available and the size of its internal cavity (∼ 6.5 Å) is optimal for the inclusion complex formation with a wide range of drugs [12]. Its molar mass is 1135 g/mol [13] and its aqueous solubility is 18.5 mg/mL [14] due to the cyclic structure, high crystal lattice energy, and formation of intramolecular hydrogen bonds [15] (Figure 2).

Given the substantial impact of an API’s solubility on its effectiveness, absorption, dissolution rate, and bioavailability, enhancing the solubility of poorly soluble drugs is crucial for ensuring their adequate therapeutic efficacy upon oral administration. The formation of inclusion complexes emerges as a potential solution to overcome limited solubility issues [16, 17].

This work was built upon the hypothesis that inclusion complexation with β-CD will not only improve DMH solubility but also mask its bitter taste [18] and that newly formed complexes will exhibit neither cytotoxic nor genotoxic effects on peripheral blood mononuclear cells (PBMCs).

We aimed to comprehensively examine the inclusion complexes of DMH and β-CD that can be used in new, improved oral pharmaceutical formulations. Increased solubility of DMH, and subsequently its bioavailability, provided by the complexation with β-CD should ensure a reduction of the dose required for therapeutic effect and, thus, a reduction of toxic and other side effects [9]. A phase solubility study was conducted to evaluate the effects of β-CD on DMH aqueous solubility in liquid state, while the characterization of formed inclusion complexes in solid state was carried out by attenuated total reflectance-Fourier transform infrared spectroscopy (ATR-FTIR) and differential scanning calorimetry (DSC). Furthermore, to ensure the safety of implementing these inclusion complexes in oral pharmaceutical formulations and to dismiss the possibility of cytotoxic or genotoxic effects after their administration, additional cytogenotoxic analyses were performed in vitro. Pure DMH, β-CD, and their inclusion complexes prepared by two different methods were tested in concentration ranges from 36.36 to 109.09 ng/mL. DMH reaches a plasma concentration of 72.6 ng/mL when it is applied in a dose of 50 mg [7], and 14.5 ng/mL when it is applied in a dose of 25 mg [19], while the concentration of 600 ng/mL is considered toxic [20]. Data regarding the cytotoxicity and genotoxicity of DMH and inclusion complexes with β-CD are lacking, therefore, these newly formed complexes were investigated by implementing the 3-[4,5-dimethylthiazol-2-yl]-2,5-diphenyl tetrazolium bromide (MTT) assay, alkaline comet assay, and cytokinesis block micronucleus cytome (CBMN-cyt) assay in human lymphocyte cultures.

## Materials and methods

### Chemicals

DMH (2-benzhydryloxy-N,N-dimethylethanamine; 8-chloro-1,3-dimethyl-7H-purine-2,6-dione, series 87081, code 3000402) was obtained as a donation from Bosnalijek, d.d. (Bosnia and Herzegovina). Kleptose^®^ (β-CD; batch E1220) was generously gifted by Roquette (France). Ethanol 96% (V/V) was purchased from Kefo^®^ d.o.o. (Bosnia and Herzegovina). Chloroform and hydrochloric acid, 37% pro analisi (p.a.), were purchased from Merck KgaA (Germany). Histopaque^®^ - 1077 (density 1.077 g/mol), a sterile, endotoxin-tested solution of polysucrose and sodium diatrizoate, was obtained from Sigma-Aldrich^®^ (St. Louis, MO, USA), as well as cytochalasin B (≥ 98%), a cell-permeable mycotoxin that inhibits cytoplasmic division by blocking the formation of contractile microfilaments, and Roswell Park Memorial Institute Medium (RPMI-1640) with L-glutamine and sodium bicarbonate. PB-MAX™ Karyotyping Medium, a fully supplemented RPMI-based medium containing fetal bovine serum, L-glutamine, and phytohemagglutinin, was purchased from GIBCO Invitrogen (Carlsbad, CA, USA). MTT (98%) reagent, dimethyl sulfoxide (DMSO, ≥ 99.7%), ethylenediaminetetraacetic acid disodium salt (Na2EDTA, 99%–101%), sodium hydroxide (NaOH, 99+%), low melting point agarose (LMPA), normal melting point agarose (NMPA), potassium chloride (KCl, 99.8%–100.5%), glacial acetic acid (99.8%), 7-imino-N,N-dimethylphenothiazin-3-amine hydrochloride (Giemsa), and 4′,6-diamidinio-2-phenylindole (DAPI, 1 mg/mL) were purchased from Sigma-Aldrich^®^ (St. Louis, MO, USA).

### Phase solubility studies

Phase solubility studies were performed according to the Higuchi and Connors’ method [21]. An excess amount of DMH was added into β-CD aqueous solutions with various concentrations (8.81–22.03 mmol/L). Sample solutions were prepared as described by Hindija et al. [22]. Solutions with concentrations of 15.86, 17.62, and 22.03 mmol/L were mixed at 50 ^∘^C for 24 h due to the limited aqueous solubility of β-CD. After the equilibrium was reached, the aliquots were filtered through a 0.2 µm pore size membrane filter (cellulose acetate filter, Sartorius, Germany), diluted with 0.1 M hydrochloric acid, and DMH concentration was determined spectrophotometrically at 277 nm (Shimadzu UV spectrophotometer-1601, Kyoto, Japan). Each measurement was run in triplicate.

Phase solubility studies, where the change of drug solubility corresponds to CD concentration, were conducted to assess the binding characteristics of the drug and CD and to determine the values of stability constant (K_s_), complexation efficacy (CE), and utility number (U_CD_). When a linear relationship between the solubility of the drug and the concentration of CD is obtained, K_s_ (M^−1^) and CE can be determined from (1) and (2): 
(1)
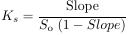

(2)
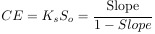

where *S*_o_ is the molar solubility (mol/L) of the drug (its aqueous solubility in the absence of β-CD) and Slope denotes the slope of the straight line (slope of the phase solubility profile).

CE values can be used to calculate the drug: cyclodextrin ratio (D:CD), according to (3) [23]: 
(3)
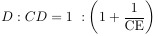


The utility number (*U*_CD_) is expressed as: 
(4)
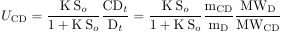

where m_D_ and m_CD_ are the drug dose and workable amount of CD in mg, respectively, while MW_D_ and MW_CD_ stand for molecular weights of D and CD [22].

### Preparation of inclusion complexes

#### Preparation of inclusion complex by kneading method

Physical mixture preparation preceded the inclusion complex formation. DMH and β-CD were accurately weighed (Analytical scale Mettler Toledo AT Balance, AT 400, Switzerland) in an appropriate 1:1 molar ratio, determined after the phase solubility analysis. Pure substances were separately pulverized. Powders were added in equivalent molar ratios, carefully blended in a glass mortar into a homogeneous mixture, and sieved through sieve No. 20 (Erweka VT/VS, Germany). The prepared physical mixture was triturated in a glass mortar with a small amount of water–ethanol solution (1:1 w/w) to obtain a homogeneous paste. The thick slurry was kneaded for 1 h, and an appropriate quantity of water–ethanol solution was intermittently added to maintain a suitable consistency. The newly formed compound was rinsed several times with a small amount of chloroform [24]. The paste was dried in a vacuum oven (Binder VD-23, Slovenia) for 6 h at 75 ^∘^C ± 0.5 ^∘^C, at a pressure of 6 ± 2 mbar. The dried mixture was pulverized into a fine powder and sieved through sieve No. 20 (Erweka VT/VS, Germany).

#### Preparation of inclusion complex by solvent evaporation method

DMH and β-CD were accurately weighed in a 1:1 molar ratio and separately dissolved in a sufficient quantity of ethanol according to their solubility using an ultrasonic bath (Bandelin Sonorex, Germany). When clear solutions were obtained, solutions of DMH and β-CD were combined and the resultant solution was stirred on a magnetic stirrer (Witeg, WiseStir MSH-20D, Germany) for 30 min at 400 rpm (30 × *g*) and 25 ^∘^C. The solvent was evaporated under vacuum at 40 ^∘^C in a rotary vacuum evaporator (Ingos RVO 200A, Czech Republic). The obtained mixture was dried in a vacuum oven (Binder VD-23, Slovenia) for 6 h at 75 ^∘^C ± 0.5 ^∘^C, at a pressure of 6 ± 2 mbar. The dried mixture was pulverized into a fine powder and sieved through sieve No. 20 (Erweka VT/VS, Germany).

### Attenuated total reflectance-fourier transform infrared spectroscopy (ATR-FTIR)

ATR-FTIR spectra of pure DMH, β-CD, their physical mixture, and inclusion complex prepared by the kneading and solvent evaporation methods were recorded using a Cary 360 FTIR (ATR) spectrophotometer, Agilent, USA. Samples were placed on a disc and the plunger was pressed tightly to hold the sample. The spectra were collected from 32 scans, recorded in the 4000–650 cm^−1^ scanning range at 4 cm^−1^ resolution.

### Differential scanning calorimetry (DSC)

DSC analysis of DMH, β-CD, their physical mixture, and inclusion complexes prepared by the kneading and solvent evaporation methods was performed using a differential scanning calorimeter DSC 204F1 Phoenix (NETZCH-Geratebau GmbH, Selb, Germany). Samples of 3–10 mg were accurately weighed, placed in 25 µL aluminum pans, and heated at a scanning rate of 10 ^∘^C/min over the temperature range from 50 ^∘^C to 400 ^∘^C. An empty pan was used as a reference standard. The measurements were carried out under dry nitrogen at a flow rate of 20 mL/min. Each run was repeated in triplicate.

### Cytotoxic and genotoxic studies

Analyses of the cytotoxic and genotoxic potential of DMH, β-CD, and their inclusion complexes prepared by the kneading and solvent evaporation methods and their impact in vitro were assessed in human lymphocyte cultures, obtained from a healthy, 37-year-old, non-smoking male volunteer. Blood was collected by venipuncture into heparinized vacutainers (BD Vacutainer Systems, Plymouth, UK), stored at room temperature, protected from light, and processed within 2 h. PBMCs were isolated by density gradient centrifugation (400 × *g*, 30 min) with separation medium (Histopaque^®^ - 1077) [25]. After isolation, PBMCs were stained with Trypan blue and counted using a hemocytometer.

#### MTT assay

The MTT assay is regarded as a sensitive and reliable colorimetric assay to quantify cellular viability, proliferation, and activation. It is based on the ability of mitochondrial nicotinamide adenine dinucleotide phosphate (NADPH)-dependent cellular oxidoreductase enzymes present in viable cells to reduce soluble tetrazolium salt (3-[4,5-dimethylthiazol-2-yl]-2,5 diphenyl tetrazolium bromide) to insoluble, colored formazan compounds [26].

DMH was administered in a concentration range from 36 to 109 ng/mL. β-CD was applied in a concentration from 0–1000 ng/mL while its highest concentration in the inclusion complex was 263.45 ng/mL. 5-fluorouracil (5-FU) at a concentration of 100 µg/mL was used as a positive control [27–30]. Untreated cells were set as negative controls and their viability was expected to remain unchanged, while the medium without cells was considered a blank to confirm the efficacy of the test procedure itself [31].

The MTT assay was conducted in triplicate in the 96-well plate, with two technical replicates. The seeding density was 6.25 × 10^3^ cells per well. Cells were incubated at 37 ^∘^C in an atmosphere of 5% CO_2_ and then treated with DMH and β-CD solutions.

After 72 h of incubation, the reagent MTT was added to samples for 3 h at 37 ^∘^C. DMSO was added to solubilize the formazan crystals followed by further incubation for 30 and 60 min at 37 ^∘^C. The optical density was measured at 570 nm using the multiplate reader Multi-Scan FC (Thermo Fisher Scientific, USA). The cell viability was determined according to (5): (5)
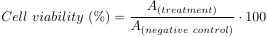



#### Alkaline comet assay

The comet assay is a sensitive technique for measuring DNA damage at the level of the individual eukaryotic cell. It is based upon the fact that denatured cleaved DNA fragments, or damaged DNA, migrate out of the cell at a different rate during electrophoresis than undamaged DNA [32]. Damaged DNA creates a “comet tail,” while the undamaged DNA remains within the cell membrane creating the “comet head.” The Comet assay is most commonly run under alkaline conditions to detect single and double-stranded DNA breaks [33].

PBMCs were isolated as previously described and then cultivated in PB-MAX™ Karyotyping Medium and incubated for 24 h at 37 ^∘^C. After incubation, PBMCs were treated with DMH, β-CD, and their inclusion complexes prepared by the kneading and solvent evaporation methods at the same concentrations as for the MTT assay. Positive and negative controls were also set up equally. Treated cells were incubated at 37 ^∘^C for 3 h and then centrifuged at 800 rpm (120 × *g*) for 5 min. The supernatant was removed and the cells were subsequently resuspended.

In a tube containing 120 µL of 0.7% LMPA, 80 µL of the sample was added for each tested concentration and controls. Prepared samples were applied to slides precoated with a 1% NMPA and covered with coverslips. After gel polymerization, coverslips were removed and slides were immersed in lysis buffer overnight at 4 ^∘^C. Afterward, slides were washed with distilled water and placed into the electrophoresis tank with an electrophoresis solution (200 mM Na_2_EDTA, 10 M NaOH, and distilled water, pH > 13) for 20 min, followed by 20 min of electrophoresis (1 V/cm). After electrophoresis, slides were gently rinsed in the following sequences: phosphate-buffered saline (PBS) for 5 min, 70% (V/V) ethanol for 5 min, and finally 96% (V/V) ethanol for 15 min.

Prior to the fluorescent microscope analysis (U-MNU2; Olympus BX51, Tokyo, Japan), slides were rehydrated and stained with DAPI (1 µg/mL). DNA damage in treated cells was evaluated using Comet Assay IV software (Instem LSS Ltd., Staffordshire, UK), by measuring tail intensity (TI%), the percent of DNA in the tail of comets. For each concentration, as well as for positive and negative controls, at least 200 comets were analyzed. The Minimum Information for Reporting Comet Assay (MIRCA) protocol was used to report the results [34].

#### Cytokinesis block micronucleus cytome (CBMN-cyt) assay

The CBMN-cyt assay is a widely used assay to evaluate cytotoxicity, DNA damage, and cytostatic effects in different tissue types [35].

In vitro analysis of the cytotoxic and genotoxic potential of DMH and its inclusion complexes with β-CD prepared by the kneading and solvent evaporation methods was performed by applying the CBMN-cyt assay in PBMCs. DNA damage events were scored specifically in once-divided binucleated (BN) cells and included micronuclei (MNi), a biomarker of chromosome breakage and/or whole chromosome loss, nucleoplasmic bridges (NPBs), a biomarker of DNA misrepair and/or telomere end-fusions, and nuclear buds (NBUDs), a biomarker of elimination of amplified DNA and/or DNA repair complexes [36]. Cytostatic and cytotoxic effects were examined by calculating the nuclear division index (NDI) and nuclear division cytotoxicity index (NDCI) based on the proportion of mono-, bi-, and multinucleated cells and necrotic and/or apoptotic cell ratios, respectively.

Whole blood, 400 µL per each treatment, was cultured in PB-MAX™ Karyotyping Medium for 72 h at 37 ^∘^C. Treatments were added to the cultures in the 25th hour of cultivation to the final concentrations of DMH 72.73, 90.91, and 109.09 ng/mL in samples with pure DMH and with complexes. Untreated cultures were set up as negative controls. Cytochalasin B was added to the final concentration of 4.5 µg/mL to block cytokinesis.

After the cultivation period, cultures were centrifuged for 10 min at 1000 rpm (188 × *g*) and subjected to hypotonic treatment with 0.56% KCl and centrifuged immediately after the hypotonic addition. Hypotonic treatment was followed by three fixations in ice-cold glacial acetic acid + ethanol (1:3) fresh fixative. The fixed lymphocyte solution was dropped on coded microscope slides. Air-dried slides were stained in 5% Giemsa for 7 min. Frequencies of MNi, NPBs, and NBUDs were observed under 400× magnification in at least 2000 BN cells for each treatment. Frequencies of mononuclear, binuclear, trinuclear, and quadrinuclear cells, as well as apoptotic and necrotic cells, were scored in a total number of at least 500 cells. All genotoxicity and cytotoxicity parameters were recognized according to the criteria given by Fenech [37, 38].

### Statistical analysis

Statistical analyses were conducted on the results of cytotoxic and genotoxic studies to identify any significant differences among the tested samples of DMH, β-CD, and their inclusion complexes at concentrations of 36.36, 54.55, 72.73, 90.91, and 109.09 ng/mL, as well as positive and negative controls. The Shapiro–Wilk test was used to assess the normality of distribution for MTT assay parameters, revealing that the data follows a normal distribution. One-way analysis of variance (ANOVA) was implemented, followed by a post-hoc Tukey–Kramer test. The normality of distribution of Comet assay parameters was examined using the Kolmogorov–Smirnov test. Afterward, the Kruskal–Wallis nonparametric test was conducted, succeeded by Dunn’s multiple comparison test. Normality of distribution for CBMN-cyt genotoxicity parameters was assessed by the Shapiro–Wilk test. Accordingly, the Kruskal–Wallis nonparametric test followed by Conover post-hoc analysis was applied to test the significance of differences between tested concentrations of DMH and its inclusion complexes with β-CD in concentrations of 72.73, 90.91, and 109.09 ng/mL and controls (negative and positive). To estimate the relationship between NDI and NDCI values in different concentrations, simple linear regression was used. Statistical analyses were performed using Microsoft Excel 2016, GraphPad Prism 8.4.3. (GraphPad Software Inc.; San Diego, CA, USA), and MedCalc^®^ v.18.9 (MedCalc bvba, Ostend, Belgium). Values were considered significantly different for *P* < 0.05.

## Results

### Phase solubility studies

**Figure 3.**
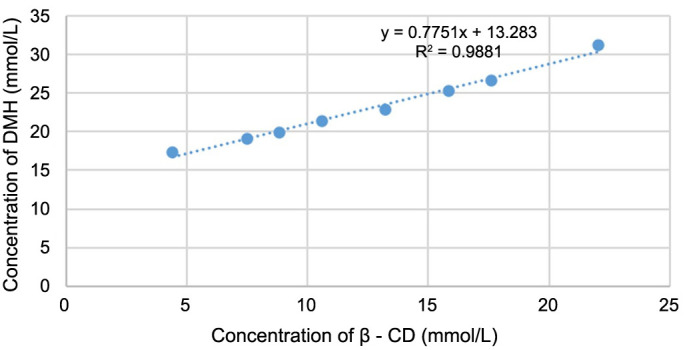
**Phase solubility diagram of DMH in an aqueous solution of β-CD.** DMH: Dimenhydrinate; β-CD: β-cyclodextrin.

The results of the conducted phase solubility analysis and determined parameters are shown in Figure 3, and Tables 1 and 2.

**Table 1:** Solubility of DMH in aqueous solutions of various concentrations of **β**-CD at 25 ^∘^C ± 0.1 ^∘^C (*n* ═ 3)

***S***	***S*_CD_ (at 25** ∘**C)**	**RSD**	***S*_CD_/*S*_o_**
4.40	17.35	0.71	0.86
7.49	19.12	0.87	0.95
8.81	19.93	0.40	0.98
10.57	21.42	1.24	1.06
13.21	22.85	1.81	1.13
15.86	25.27	0.60	1.25
17.62	26.68	0.96	1.32
22.03	31.15	0.91	1.55

DMH: Dimenhydrinate; β-CD: β-cyclodextrin; *S*: Concentration of β-CD; *S*_CD_: Concentration of DMH in CD solution (mmol/L); RSD: Relative standard deviation; *S*_CD_/*S*_o_: Solubility enhancement factor calculated as the ratio of drug solubility in CD solution (*S*_CD_) versus drug solubility value (*S*_o_) measured in the absence of CD.

**Table 2:** Essential parameters for complexation of DMH and β-CD determined by phase solubility analysis

**Slope^a^**	***R* ^2a^**	***K_s_* (M∘1)^b^**	**CE^c^**	**Dose^d^ (mg)**	**D:CD^e^**	***U*_CD_^f^**
0.7751	0.9881	171.10	3.45	25	1:1.29	0.214

^a^Values obtained directly from the drug phase solubility diagram; ^b^The K_s_ calculated from the slope of phase solubility diagram according to Equation (1); ^c^Complexation efficiency calculated from the slope of phase solubility diagram according to Equation (2); ^d^Oral dosage of DMH for children; ^e^The drug: Cyclodextrin molar ratio is based on the calculated complexation efficiency according to Equation (3); ^f^The utility number was calculated according to Equation (4). The *U*_CD_ was calculated for the values of *K*_s_, *S*_o_ for DMH determined at 25 ^∘^C. The presented results for the *U*_CD_ are related to the working concentration of 1% CD. DMH: Dimenhydrinate; β-CD: β-cyclodextrin; *K*_s_: Stability constant; CE: Complexation efficiency; D: Drug; *U*_CD_: Utility number; CD: Cyclodextrin.

### Attenuated total reflectance-fourier transform infrared spectroscopy (ATR-FTIR)

**Figure 4.**
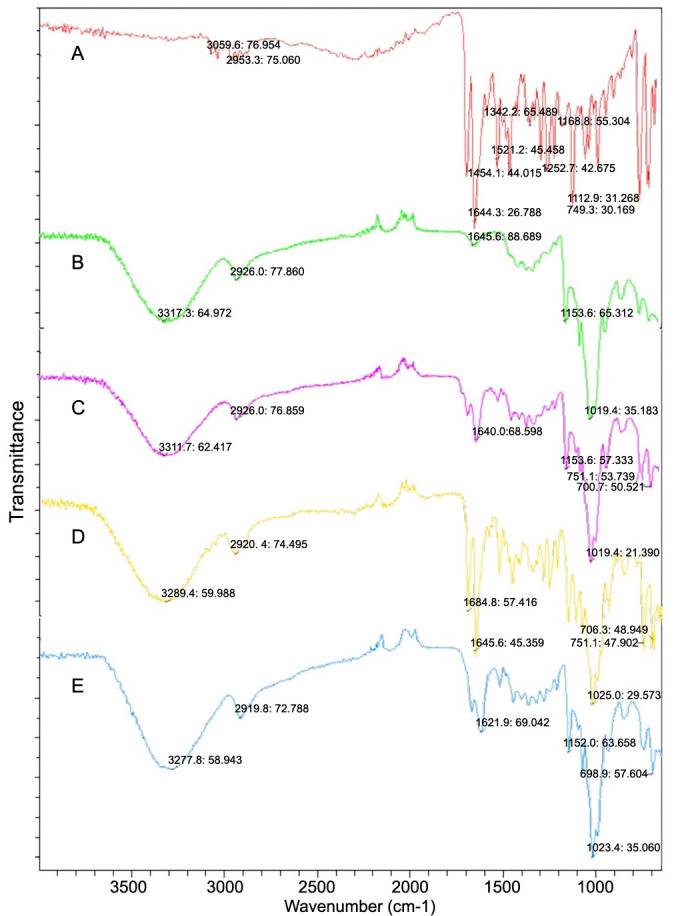
**Illustrating the FTIR spectra of pure DMH (A), pure β-CD (B), their physical mixture (C), inclusion complex of DMH and β-CD prepared by the kneading method (D), and inclusion complex of DMH and β-CD prepared by the solvent evaporation method.** DMH: Dimenhydrinate; β-CD: β-cyclodextrin.

The ATR-FTIR spectra of binary systems (physical mixture and inclusion complexes prepared by kneading method and solvent evaporation method) were compared to those of the pure substances (DMH and β-CD) (Figure 4). It was investigated whether characteristic bands of the pure substances changed, which would indicate the existence of a complex as a new compound with different spectroscopic bands.

A comparison between the intensity of FTIR signals of pure DMH and its physical mixture and inclusion complexes prepared by the kneading method and the solvent evaporation method with β-CD is given in Table 3. A comparison between the intensity of FTIR signals of pure β-CD and its binary systems with DMH (physical mixture and inclusion complexes prepared by the kneading method and the solvent evaporation method) is given in Table 4. The tables include calculated chemical shift changes (|Δ δ|) in the samples and transmittance expressed in percentage.

**Table 3:** Comparison of the intensity of FTIR signals of pure DMH and binary systems with β-CD (physical mixture, inclusion complex prepared by kneading method, and inclusion complex prepared by solvent evaporation method)

**Parameter**	**Sample**	**Functional group**			
		****ν**[–NH_2_ and –NH]**	****ν**[C=O]**	****ν**[C=C aromatic]**	****ν**[C–Cl carbonyl]**
Wavenumber (cm^−1^)	DMH	3059.6	1644.3	1112.9	749.3
	P.M.	overlapped	1645.6	1114.1	751.1
	Dβ-K	overlapped	1689.0	1074.2	751.1
	Dβ-SE	overlapped	1682.7	1067.5	698.9
Changes |Δ δ|	P.M.	/	1.3	1.2	1.8
	Dβ-K	/	44.7	38.7	1.8
	Dβ-SE	/	38.4	45.4	50.4
Transmittance (%)	DMH	76.95	26.79	31.27	30.17
	P.M.	n.o.^*^	45.36	67.20	47.90
	Dβ-K	n.o.^*^	82.41	56.72	53.74
	Dβ-SE	n.o.^*^	76.13	68.36	57.60

|Δ δ| ═ δ_(binary system)_ − δ_(pure DMH)_; ^*^The peak of the functional group was not observed in the binary system, and the percentage of transmittance could not be read. P.M.: Physical mixture; Dβ-K: Inclusion complex of DMH and β-CD prepared by the kneading method; Dβ-SE: Inclusion complex of DMH and β-CD prepared by the solvent evaporation method; DMH: Dimenhydrinate.

**Table 4:** Comparison of the intensity of FTIR signals of pure β-CD and binary systems with DMH (physical mixture, inclusion complex prepared by kneading method, and inclusion complex prepared by solvent evaporation method)

**Parameter**	**Sample**	**Functional group**			
		****ν**[OH]**	****ν**[–CH_2_ and –CH]**	****ν**[H–O–H]**	****ν**[C–O–C]**
Wavenumber (cm^−1^)	β-CD	3317.3	2926.0	1645.6	1019.4
	P.M.	3311.7	2926.0	1640.8	1019.4
	Dβ-K	3289.4	2920.4	1648.8	1025.0
	Dβ-SE	3277.8	2919.8	1621.9	1023.4
Changes |Δ δ|	P.M.	5.6	0	4.8	0
	Dβ-K	27.9	5.6	39.5	5.6
	Dβ-SE	39.5	6.2	23.7	4.9
Transmittance (%)	β-CD	64.97	77.86	88.69	35.18
	P.M.	62.42	76.86	68.60	21.39
	Dβ-K	59.97	74.50	57.41	29.57
	Dβ-SE	58.94	72.79	69.04	35.06

|Δ δ| ═ δ_(binary system)_ - δ_(pure β-CD)_. P.M.: Physical mixture; Dβ-K: Inclusion complex of DMH and β-CD prepared by the kneading method; Dβ-SE: Inclusion complex of DMH and β-CD prepared by the solvent evaporation method; β-CD: β-cyclodextrin.

### Differential scanning calorimetry (DSC)

**Figure 5.**
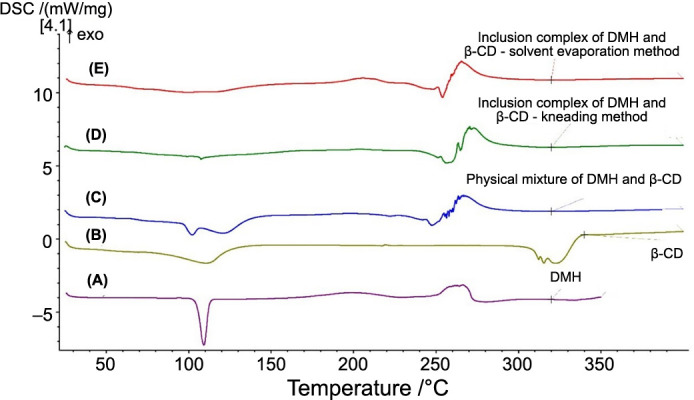
**Illustrating the DSC thermograms of pure DMH (A), pure β-CD (B), their physical mixture (C), inclusion complex of DMH and β-CD prepared by the kneading method (D), and inclusion complex of DMH and β-CD prepared by the solvent evaporation method (E).** DMH: Dimenhydrinate; β-CD: β-cyclodextrin; DSC: Differential scanning calorimetry.

DSC analysis was carried out to examine the behavior of individual components, their physical mixture, and inclusion complexes prepared by the kneading method and the solvent evaporation method during heating (Figure 5), such as crystallization, phase transformation, dehydration, and decomposition.

### MTT assay

**Figure 6.**
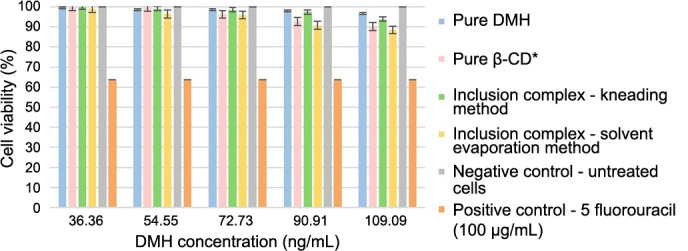
**Illustrating thepercentage of cell viability in human PBMCs after treatment with pure DMH, pure β-CD, and their inclusion complexes prepared by the kneading and solvent evaporation methods.** *Pure β-CD was applied in concentrations of 0, 250, 500, 750, and 1000 ng/mL. DMH: Dimenhydrinate; β-CD: β-cyclodextrin; PBMCs: Peripheral blood mononuclear cells.

Stock solutions of DMH and its inclusion complexes with β-CD prepared by the kneading and solvent evaporation methods in RPMI-1640 Medium and PB-MAX™ Karyotyping Medium were diluted and applied as treatments with five increasing concentrations, together with blank, positive, and negative controls. The results of cell viability in human PBMCs in the presence of pure DMH, pure β-CD, and their inclusion complexes are shown in Figure 6.

ANOVA analysis showed a significant difference between administered treatments (*P* < 0.05). Post-hoc Tukey–Kramer test showed there were no statistically significant differences between the negative control and treatments with pure DMH. However, statistically significant differences were observed for pure β-CD and inclusion complexes prepared by the kneading method and the solvent evaporation method. Negative control and all treatments were significantly different from the positive control. Another analysis was conducted to establish which concentration of pure β-CD and inclusion complexes prepared by the kneading and solvent evaporation methods differed from the negative control. A statistically significant difference between treatments and the negative control was recorded only for the highest concentrations. For the lower concentration of treatments, there were no significant differences compared to the negative control.

### Alkaline comet assay

Genotoxic effects of DMH, pure β-CD, and their inclusion complexes prepared by the kneading and solvent evaporation methods were expressed as a percent of DNA in the tail of comets (tail intensity [TI]), based on the analysis of 205 comets for each concentration (Table 5).

**Table 5:** Tail intensity (%) in PBMCs exposed to different concentrations of the tested treatments (mean ± SD)

**Concentration of DMH (ng/mL)**	**Pure DMH**	**Inclusion complex kneading method**	**Inclusion complex solvent evaporation method**
36.36	2.51 ± 1.77	3.10 ± 1.28	1.79 ± 2.21
54.55	2.83 ± 1.54	3.04 ± 1.28	1.92 ± 2.23
72.73	3.44 ± 1.13	2.95 ± 1.43	2.53 ± 1.72
90.91	3.47 ± 1.08	2.88 ± 1.63	2.75 ± 1.27
109.9	3.59 ± 0.79	2.79 ± 1.55	3.57 ± 0.99
**Concentration of β-CD (ng/mL)**	**Pure β-CD**	**Negative control**	**Positive control 5-FU (100 µg/mL)**
250	3.02 ± 1.45	3.22 ± 1.21	5.93 ± 0.23
500	3.22 ± 1.09	/	/
750	3.25 ± 1.02	/	/
1000	3.42 ± 1.08	/	/

DMH: Dimenhydrinate; β-CD: β-cyclodextrin; PBMCs: Peripheral blood mononuclear cells; 5-FU: 5-fluorouracil.

TI was not significantly increased for any of the treatments in comparison with the negative control (untreated cells). All treatments, as well as the negative control, were significantly different from the positive control (cells treated with 5-FU).

Comet images recorded for treatments of PBMCs with pure DMH, pure β-CD, and their inclusion complexes prepared by the kneading and solvent evaporation methods at the highest concentrations (for DMH and inclusion complexes 109.09 ng/mL, and for pure β-CD 1000 ng/mL) are shown in Table 6.

**Table 6:** Representative comet images from treatment of PBMCs with pure DMH, pure β-CD, and their inclusion complexes prepared by the kneading and solvent evaporation method

Negative control	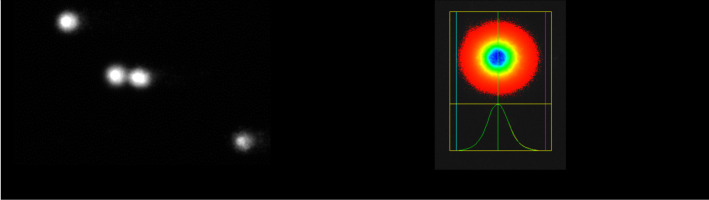
Positive control	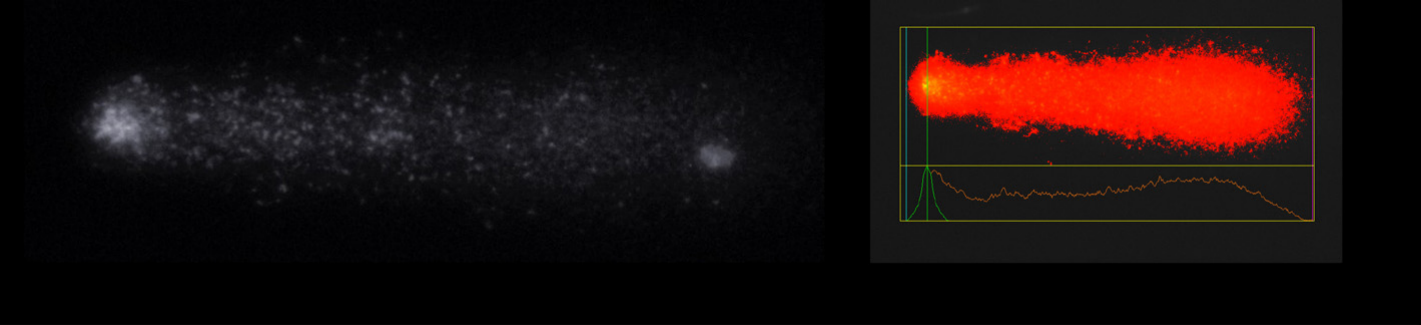
DMH	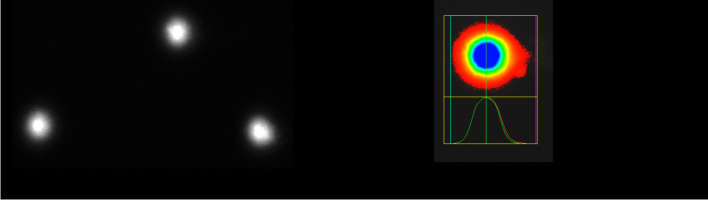
β-CD	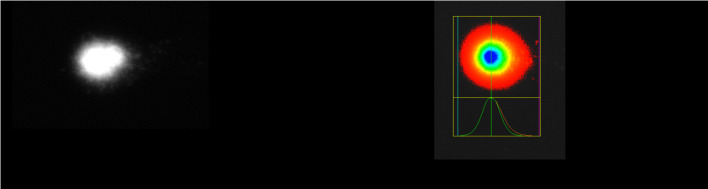
Inclusion complex - kneading method	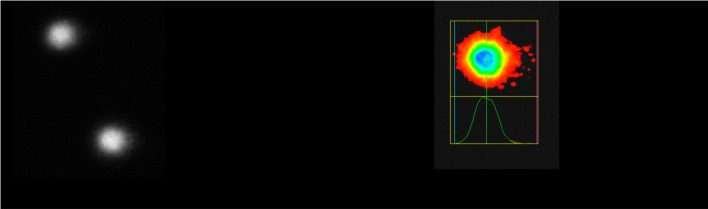
Inclusion complex - solvent evaporation method	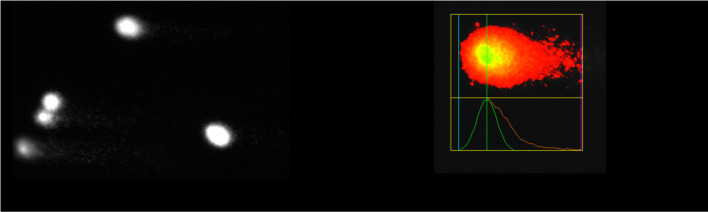

DMH: Dimenhydrinate; β-CD: β-cyclodextrin; PBMCs: Peripheral blood mononuclear cells.

### Cytokinesis block micronucleus cytome (CBMN-cyt) assay

Results of the CBMN-cyt assay are presented in Table 7 and show a significant decrease (*P* ═ 0.002) in the total MNi frequency in all treatments with the inclusion complex prepared by the solvent evaporation method and in treatment with pure DMH at the lowest concentration (72.73 ng/mL) compared to the negative control. For these treatments, the same results were obtained for the frequency of BN cells with MNi (*P* ═ 0.001). The frequency of NBUDs was significantly decreased in all treatments compared to the positive control (*F* ═ 2.725; *P* ═ 0.015), while for the frequency of NPBs and values of NDI and NDCI, significant differences were not found.

**Table 7:** Results of CBMN-cyt biomarkers frequency and NDI and NDCI values in treatments with pure DMH, inclusion complexes prepared by the kneading method (Dβ-K), and the solvent evaporation method (Dβ-SE) (mean ± SD)

**Treatments^t^**	**Total MNi**	**BN cells with MNi**	**NBUDs**	**NPBs**	**NDI**	**NDCI**
Negative control	7.5 ± 2.38	7.25 ± 2.41	0.75 ± 0.5^s^	0.5 ± 0.58	1.576 ± 0.10	1.569 ± 0.10
DMH 1	4.25 ± 0.96^r, s^	4 ± 1.25^a^	0.75 ± 0.96^s^	0.25 ± 0.5	1.605 ± 0.10	1.600 ± 0.09
DMH 2	6.5 ± 1.92^s^	5.75 ± 1.92	1.75 ± 1.26^s^	0.25 ± 0.5	1.728 ± 0.10	1.718 ± 0.10
DMH 3	6.5 ± 2.38^s^	6.5 ± 2.34	1 ± 0.82^s^	0.25 ± 0.5	1.674 ± 0.12	1.667 ± 0.12
Dβ-K 1	6.25 ± 2.22^s^	6 ± 2.18	1.75 ± 0.5^s^	0.25 ± 0.5	1.704 ± 0.10	1.699 ± 0.11
Dβ-K 2	9 ± 4.08^s^	8.5 ± 3.45	0.75 ± 0.96^s^	0.5 ± 0.58	1.770 ± 0.06	1.759 ± 0.06
Dβ-K 3	8.25 ± 3.20^s^	8.25 ± 3.06	0.75 ± 0.5^s^	0.5 ± 0.58	1.775 ± 0.13	1.768 ± 0.13
Dβ-SE 1	2.75 ± 2.63^r, s^	2.75 ± 2.15^r^	1.25 ± 0.96^s^	0	1.617 ± 0.08	1.613 ± 0.08
Dβ-SE 2	3.5 ± 0.58^r, s^	3.5 ± 1.04^r^	0.25 ± 0.5^s^	0.25 ± 0.5	1.635 ± 0.19	1.628 ± 0.19
Dβ-SE 3	3.5 ± 2.08^r, s^	3.25 ± 1.61^r^	1.25 ± 1.5^s^	0.25 ± 0.5	1.570 ± 0.17	1.564 ± 0.17
Positive control	19.25 ± 6.65	19 ± 6.56	3.5 ± 1.92	1 ± 0.82	1.612 ± 0.01	1.577 ± 0.01

^r^Significant decrease (*P* < 0.005) compared to negative control; ^s^Significant decrease (*P* < 0.005) compared to positive control; ^t^Concentration (ng/mL) of pure DMH and DMH in complexes were 1 (72.73), 2 (90.91), and 3 (109.09). DMH: Dimenhydrinate; CBMN-cyt: Cytokinesis-block micronucleus cytome; BN: Binucleated; MNi: Micronuclei; NPBS: Nucleoplasmic bridges; NBUDs: Nuclear buds; NDI: Nuclear division index; NDCI: Nuclear division cytotoxicity index.

## Discussion

The complexes were prepared using two different methods: the kneading method and the solvent evaporation method. Both methods involved the use of adequate organic solvents to ensure that all or at least a small part of the components are brought into the solution, allowing secondary bonds to form between the molecules, which provide the formation of inclusion complexes with proper stability [39]. The kneading method is simple, efficient, and provides a good yield of formed inclusion complexes. It is convenient for the preparation of complexes with poorly soluble drugs because of drug dispersion during complex formation [40, 41]. The solvent evaporation method is simple, economical, and suitable for large-scale preparation of complexes [42]. To the best of our knowledge, this topic has not yet been investigated in any published studies. Complexes of DMH were prepared either with different hydrophilic derivatives of β-CD [43] or with different methods [44, 45]. However, the effects on the viability of human lymphocytes have not been explored yet.

A-type phase solubility isotherms are characteristic of water-soluble complexes. The phase solubility diagram in this study revealed an A_L_ type isotherm, indicating that the solubility of DMH linearly increased with increasing β-CD concentration (Figure 3). The slope, defined as the change in *y*-axis (concentration of DMH) for a one-unit increase in the *x*-axis (β-CD concentration), indicates the steepness of a regression line. If one molecule of a drug forms a complex with one molecule of CD, the slope of a straight line is less than unity, and the value of *K*_S_ can be calculated by applying equation (1). In this case, the slope value was 0.7751, implying the linear DMH solubility enhancement in β-CD solution and the formation of a 1:1 complex.

Optimal *K*_s_ values range from 100 to 5000 M^−1^. Lower values imply very labile complexes with premature drug release and insignificant solubility improvement, while higher values imply very stable complexes with incomplete or obstructed drug release from the CD cavity [10]. CE determination, reliant on the phase-solubility profile slope, is a suitable parameter for evaluating the solubilizing potential of CDs and is a less variable indicator than *K*_s_, which is influenced by the intercept and intrinsic solubility affected by formulation excipients. In this study, the values of *K*_s_ (171.10 M^−1^) and CE (3.45) indicated the formation of a stable complex with expected appropriate drug release and satisfying solubility improvement. Solubility enhancement factor values (Table 1) indicate that notable solubility enhancement is attained in solutions with a β-CD concentration of 15.86 mmol/L and above.

Values of the dimensionless number, U_CD_ ≥ 1, implicate adequate solubilization provided by complexation with CDs. Values ≥ 1 indicate sufficient solubilization through CD complexation, while values below 1 signify incomplete solubilization [46]. The calculated *U*_CD_ value for 1% (w/w) β-CD concentration was < 1 implying that it was insufficient for complete solubilization of 25 mg DMH/mL water. A concentration of 1.8% (w/w) would be required to adequately provide the dissolution of 25 mg DMH/mL of water. In that case, the *U*_CD_ value is 1.156. The D:CD ratio, based on the CE values, equaled 1:1.29 and confirmed the formation of a 1:1 inclusion complex.

In the ATR-FTIR spectrum of pure DMH (Figure 4A), the characteristic peak at 3059.6 cm^−1^ corresponded to its amino groups. A sharp, stretched peak at 1644.3 cm^−1^ was for C=O stretching, at 1112.9 cm^−1^ was the peak for C=C stretching of the aromatic rings, and at 749.3 cm^−1^ was the peak for C–Cl stretching of the carbonyl chloride [5, 47].

In the spectrum of pure β-CD (Figure 4B), a peak at 3317.3 cm^−1^ represents the vibration of symmetrical and asymmetrical stretching of primary hydroxyl (–OH) groups on C6 atoms of glucose molecules, located on the narrower side of the β-CD ring, and another peak at 2926.0 cm^−1^ showed the vibration of –CH and –CH_2_ groups. The peak at 1645.6 cm^−1^ may be attributed to residual H–O–H molecules and –OH groups in the glucose moieties of β-CD. The peak at 1153.6 cm^−1^ originated from an ether-like bond between cyclically linked glucose molecules of β-CD. A distinct, sharp peak at 1019.4 cm^−1^ was assigned to the C–O–C stretching vibrations [6, 48, 49].

In the physical mixture of DMH and β-CD (Figure 4C), the peak of DMH originating from its amino groups was overlapped by the peak of β-CD ascribed to –OH stretching vibrations. The peaks ascribed to –CH_2_ and –CH, H–O–H, and C–O–C bending vibrations of β-CD and the peaks corresponding to C=O and C–Cl stretching vibrations of DMH were shifted as shown in Table 3. The peak at 751.1 cm^−1^ is a result of the deformation vibrations outside of the plane (δ) and flexion of –CH bonds of the aromatic core. Peaks characteristic of pure substances that were still observed in the spectrum of the physical mixture imply that the DMH molecule was not entirely embedded into the β-CD cavity.

The spectra of inclusion complexes of DMH and β-CD prepared by the kneading method (Figure 4D) and solvent evaporation method (Figure 4E) differ from the spectra of pure substances (Figure 4A and 4B) and their physical mixture (Figure 4C). The shifts and intensity changes of the peak of pure β-CD ascribed to the –OH, –CH, and –CH_2_ stretching vibrations are shown in Table 4. The peak of DMH ascribed to the amino group was not identified, while peaks associated with the C=C stretching of the aromatic rings, C=O, and C–Cl stretching in the DMH molecule had different wavelengths and intensities in the spectra of inclusion complexes (Table 3). Embedding of the DMH molecule into the central cavity of β-CD can also be confirmed due to significant changes in the intensity and wavelength of the peak characteristic for H–O–H stretching vibrations, indicating that water molecules were shifted out of the cavity by DMH molecules. Insertion of the benzene ring part into the electron-rich cavity of β-CD increased the density of the electron cloud, leading to frequency changes. Different frequencies of peaks in the inclusion complexes compared to the pure molecules appeared due to the changes in the microenvironment caused by hydrogen bond formation and Van der Waals forces. The penetration of the guest molecule that occurs when the inclusion complex is formed causes consequent structural rearrangement of the H-bonded scheme in the host’s inner cavity, which can be proven by changes in specific peaks’ shapes, positions, and intensities with respect to the pure compounds and physical mixture [50].

DSC is one of the best tools to confirm complex formation by the disappearance of the characteristic endothermic peaks of the drug in the thermogram of the formed inclusion complex [51]. When guest molecules are embedded in the CD cavity, their melting, boiling, or sublimation point generally could shift to a different temperature or disappear within the temperature range where CD decomposes [52].

Figure 5A presents the DSC thermogram of pure DMH. A sharp, prominent endothermic peak at 106.2 ^∘^C appeared at its melting point [53]. Decomposition of the drug occurred at temperatures above 250 ^∘^C [54]. The DSC thermogram of pure β-CD (Figure 5B) showed an endothermic peak at 110.8 ^∘^C attributed to the liberation of crystal water from β-CD. A small peak observed at 235 ^∘^C may be due to the glass transition, and finally, the degradation of β-CD was at 322.5 ^∘^C [55, 56].

The DSC curve of the physical mixture of DMH and β-CD (Figure 5C) showed endothermic peaks at 102.1 ^∘^C (melting point of DMH), 120.8 ^∘^C (dehydration of β-CD), and 247.4 ^∘^C (decomposition of DMH). The slight changes observed in the melting endotherm for DMH (decreased temperature and intensity) indicated that there was a weak interaction between DMH and β-CD in a simple physical mixture.

In the thermograms of inclusion complexes of DMH and β-CD prepared by the kneading method (Figure 5D) and solvent evaporation method (Figure 5E), the peak characteristic of the melting point of DMH disappeared, indicating successful inclusion complexation of DMH into the central cavity of β-CD. The formation of an inclusion complex was suggested not only by the absence of the melting endotherm of DMH but also by the reduction of the dehydration curve in the inclusion complex prepared by the kneading method (Figure 5D) compared to the physical mixture (from 120.8 ^∘^C to 107.4 ^∘^C). This implies the displacement of water molecules by DMH. The endothermic peak corresponding to the dehydration of β-CD was not identified in the inclusion complex prepared by the solvent evaporation method (Figure 5E). The complete disappearance of DMH’s endothermal peak can be assumed as proof of interactions with β-CD. This can be considered an indication of drug amorphization and/or inclusion complex formation. The disappearance of a sharp endothermic peak in the range of the decomposition of pure DMH is due to its encapsulation in the host’s inner cavity.

The results of the MTT assay led to the conclusion that applied treatments with DMH, β-CD, and their inclusion complexes did not reduce cell viability because there were no significant differences compared to the negative control. Only the treatments with the highest concentrations of pure β-CD (1000 ng/mL) and inclusion complexes prepared by both methods (DMH concentration 109.09 ng/mL), which are considerably higher than therapeutic concentrations (72.6 ng/mL), were significantly different from the negative control. Recorded cell viability for these concentrations was 90.3% for β-CD, 91.1% for the inclusion complex prepared by the kneading method, and 85.8% for the inclusion complex prepared by the solvent evaporation method. Cell viability of the negative control was considered to be 100% because those cells were untreated. Recorded cell viability for the positive control was 64.8%, which significantly differed from the negative control and all treatments (*P* < 0.05). According to Gokarn et al. [57], DMH may slightly affect the viability of HEK293 cells. Previous similar studies reported that β-CD does not affect cell viability in HeLa cells [58], LO2 cells [59], endothelial (HUVEC) cells [60], Calu-3 cells [61], mouse retinal cells [62], or HCT-116 and MDA-MB-231 cancer cells [63]. This leads to the consideration of these complexes as non-cytotoxic or with low cytotoxic potential in the therapeutic range of dosage. However, the results of the MTT assay solely indicate cell viability, without distinguishing between cytotoxic, cytostatic, or antiproliferative effects [64], thus complementary assays were conducted to aid in data analysis and interpretation alongside the MTT assay.

According to the presented results of the alkaline comet assay, DMH, β-CD, and their inclusion complexes prepared by the kneading and solvent evaporation methods did not show genotoxic effects on PBMCs. The percentage of DNA in the comet tail did not significantly differ (*P* > 0.05) from the negative control after treatment at various concentrations. Treatments with inclusion complexes of DMH and β-CD prepared by the solvent evaporation method at concentrations of 36.36, 54.55, 72.73, and 90.91 ng/mL even exhibited a significantly lower percentage of DNA in the comet tail compared to the negative control (*P* < 0.05). For 5-FU, genotoxic effects were observed due to an increase in migration (mean tail length) of cell DNA compared to the untreated cells in the negative control (*P* < 0.05). The mean value of tail intensity for the positive control was 61.6%, while for the negative control it was 12.3%. Mean % tail DNA values for all the treatments at various concentrations ranged from 6.7%--14.5%, respectively (Table 5). This implies that there were no significant increases in the induction of DNA strand breaks in the PBMCs at any dose compared to the negative control. DNA integrity of different inclusion complexes of β-CD and its hydrophilic derivative hydroxypropyl-β-CD (HP-β-CD) was examined by comet assay in cell line HL-60 [65], dermal fibroblasts of healthy subjects [66] or Niemann-Pick C1 patients [67], differentiated human macrophage-like THP1 cells [68], Jurkat cells (ATCC, clone E6-1) [69], human lymphocytes [70], and human leucocytes [71] and found to be safe, with no genotoxic effects shown. It is also reported that DMH did not cause DNA strand breaks in rat primary hepatocytes [72].

No significant differences were found in the CBMN-cyt assay biomarker analysis that would indicate genotoxic effects of all tested complexes. The frequencies of observed MNi, NBUDs, and NPBs in all applied concentrations were not significantly increased compared to the negative control. It was even noted that the frequency of total MNi as well as BN cells with MNi significantly decreased after treatment with complexes prepared by the solvent evaporation method in all concentrations (*P* < 0.005), which definitely classifies this compound as non-genotoxic in normal PBMCs. Inclusion complexes of β-CD and its hydrophilic derivatives (HP-β-CD, methyl-β-CD, and sulfobutyl ether-β-CD) do not affect the frequency of genotoxicity biomarkers in human lymphocytes [73], THP1 cells [68], or Chinese hamster ovary-K1 cells [74]. The genotoxic effects of inclusion complexes of β-CD were also evaluated in vivo, revealing no evidence of induced genetic damage [75, 76]. As expected, a significant increase in all biomarkers was found in the positive control. The cytotoxic and cytostatic potential was not determined for any of the tested complexes because no significance was found for the NDI and NDCI values between treatments and negative control. Considering the low cytotoxicity rate and high viability of PBMCs assessed by the MTT assay, these results confirmed that the tested complexes do not have aneugenic or clastogenic effects in normal PBMCs. Although PBMCs offer numerous advantages regarding their accessibility and storage convenience [77], considering the absence of enzyme systems responsible for metabolic activation [78], observed cytotoxic and genotoxic effects could be perceived as relevant within the framework of this model. The advantage also lies in the use of primary PBMC culture regardless of the mentioned metabolic activation, which was not primarily necessary in this case.

The specificity of the genotoxicity assay may depend more on the particular test system used rather than the presence or absence of metabolic activation [79]. Gokarn et al. [57] examined the cytotoxicity of DMH by MTT assay and metabolic activation was not performed. Another study reported that DMH’s metabolite, diphenhydramine, did not induce chromosomal aberrations in cultured human lymphocytes or fibroblasts in the absence of exogenous metabolic activation [80].

PBMCs are used in genotoxicity testing and metabolic studies, but the use of metabolic activation with PBMCs depends on the specific research context, primarily on the research objectives and the compounds being tested.

The use of metabolic activation to simulate the metabolic processes that occur in the body and assess the genotoxic potential of compounds that require metabolic transformation to become genotoxic may not always be necessary with PBMCs, especially in studies focusing on the immediate metabolic response of PBMCs or assessing metabolic flexibility and capacity [81]. Therefore, PBMCs can be used both with and without metabolic activation in different research contexts, depending on the specific goals of the study and the need to evaluate the genotoxic effects of compounds that require metabolic activation for their activity [82].

The limitation of the study is working on only one type of cells and solely on an in vitro model of primary human healthy cell culture (short-term), rather than on additional specific cell lines or some in vivo model. Additional research should involve a significantly larger number of in vitro cell models with preserved metabolic functions to validate these effects.

## Conclusion

The analyses in this research indicate the formation of a new, stable inclusion complex between DMH and β-CD in a 1:1 stoichiometric ratio. Previously uninvestigated complex formation was confirmed by ATR-FTIR and DSC analyses according to the changes in ATR-FTIR spectra and the absence of characteristic endothermic peaks of DMH and β-CD in DSC thermograms of inclusion complexes. It was proven that both the kneading and solvent evaporation methods successfully provided complexation.

Prior studies did not assess the cytotoxic and genotoxic potential of DMH-β-CD inclusion complexes. Our findings from the MTT assay, comet assay, and CBMN-cyt assay indicate no observed cytotoxic or genotoxic effects on normal PBMCs across five concentrations, two of which exceeded therapeutic levels. Given the absence of metabolic activation enzyme systems in lymphocytes, the observed cytotoxic and genotoxic effects might be relevant within this model, necessitating further research with more in vitro cell models to validate them.

Inclusion complexation with β-CD might be an efficient approach to overcome the poor solubility issues of DMH and to mask its bitter taste. It can be further investigated whether the newly formed, non-toxic complexes enhance the physicochemical properties of DMH in pharmaceutical formulations and if so, the DMH-β-CD system can be used in new drug delivery systems.
